# Early weaning and biological sex shape long-term immune and metabolic responses in pigs

**DOI:** 10.1038/s41598-023-42553-9

**Published:** 2023-09-23

**Authors:** Mahsa Fardisi, Kyan Thelen, Allegra Groenendal, Mrigendra Rajput, Kimberly Sebastian, G. Andres Contreras, Adam J. Moeser

**Affiliations:** 1 Department of Large Animal Clinical Sciences, College of Veterinary Medicine, Michigan State University, East Lansing, MI 48824 USA; 2 Department of Pathology and Diagnostic Investigation, College of Veterinary Medicine, Michigan State University, East Lansing, MI 48824 USA

**Keywords:** Developmental biology, Immunology, Inflammation, Animal physiology

## Abstract

During the early pre and postnatal life, host and environmental factors can impart a major influence on immune development, thus shaping lifelong disease resistance. Two major factors known to influence immune function and mortality in animals and people are early life stress and biological sex. How these two factors interact to shape long-term immune development and later life disease risk is poorly understood. Here we investigated how early weaning, a common early life stressor in pigs, and biological sex impacts long-term systemic inflammatory responses and hypothalamic–pituitary–adrenal axis (HPA axis) activation later in life. Ten-week-old female (F), intact-male (IM) and castrated-male (CM) pigs that were randomly assigned to early weaning (EW) and later weaning (LW) (at 15 or 28 days of age, respectively) were intramuscularly injected with either saline vehicle or lipopolysaccharide (LPS) to induce a systemic inflammatory response. Complete blood counts (CBC), proinflammatory cytokines, cortisol, testosterone, estradiol, and rectal temp were measured at 0 h, 2 h, and 4 h post-LPS challenge. At 4 h post-LPS, peritoneal fluid (PF) and white blood cells (WBC) were collected for differential analysis. LPS challenge significantly increased rectal temp and plasma cortisol level in all treatment groups. Together, the CBC results and immune cell counts in peritoneal cavity indicated that EW-F exhibited greater systemic immune response characterized by increased neutrophils to lymphocytes ratio (NLR) and enhanced neutrophil trafficking to the peritoneal cavity. Early weaning had an opposite effect on IM and CM pigs, which exhibited a suppressed LPS-induced neutrophil migration. Early weaning induced significantly greater cortisol responses only in IM pigs indicating a heightened HPA axis responses in EW-IM. how early weaning and biological sex affect immune and stress responses in pigs. Together, these results demonstrate that early weaning and biological sex and castration shape later life immune responses in pigs and provides insight into potential mechanisms driving sex differences in later life inflammatory disease risk and mortality.

## Introduction

Early weaning is a management practice in swine production systems, where piglets are weaned from their dam at an early age commonly between 15 and 21 days of age, to optimize the reproductive efficiency of the breeding herd and overall productivity^1^. While early weaning is necessary from an economic standpoint, this practice is associated with increased stress on piglets (e.g. maternal and littermate separation, change in environment and food source, and exposure to new antigens and pathogens), and thus heightened disease risk in piglets^2–4^. This is in stark contrast to the natural setting in wild pigs who are weaned gradually at around 3–4 months of age. Despite the widespread adoption of early weaning, the mechanisms contributing to the increased disease risk in early-weaned pigs remain largely unknown.

The psychosocial and environmental stressors that are initiated by early weaning in piglets occur during a critical period of immune system plasticity and maturation, and thus can have long-lasting programming effects on long-term immune function and disease resistance. A similar and more well-studied paradigm exists in people, as early life stress or adverse childhood events (ACEs) are linked to greater disease susceptibility in humans^5,6^, further underscoring the importance of understanding early-life stressors in animal models to potentially inform human health research. We have demonstrated previously that early weaning in piglets cand alters development and function of the intestinal epithelial barrier and nutrient transport functions and induce lasting changes in the enteric nervous system^7–10^. We also showed that early weaning causes intestinal mast cell numbers and activity. However, the impact of early weaning on systemic immune development and responses to later life immune challenges has not been defined.

A critical, yet understudied factors impacting stress responses and disease risk in pigs is biological sex. Biological sex is known to play a significant role in determining the outcome of various infectious diseases across species^11^. In pigs, castrated male pigs have been reported to exhibit higher mortality rates than females, with rates up to 2.5 times higher^12,13^. This phenomenon parallels observations in humans and other species where sex differences in mortality and morbidity rates are well-documented, often with males at increased risk for mortality while females may be more prone to inflammatory and autoimmune diseases^14,15^. Despite the critical role of sex as a determinant of disease risk, it remains vastly understudied, particularly in production animals such as the pig. Exploring how early weaning and sex influence immune system responses to stress and pathogen challenges is an underexplored yet critical area of investigation. The primary objective of this study is to determine how early weaning impacts long-term systemic immune and neuroendocrine responses to later life challenges in pigs and to assess whether biological sex and castration impact this response.

## Material and methods

### Handling guidelines for animals

All procedures involving animals for this research were approved by and under the supervision of the University Institutional Animal Care and Use Committee (IACUC) (Protocol #: PROTO201900090). The welfare of the animals was given utmost priority throughout the entire research process, including housing, handling, and experimental procedures.

### Confirmation of method compliance with guidelines

We affirm that all methods employed in this study were conducted in accordance with the relevant IACUC and ARRIVE guidelines and regulations for animal research. The research adhered to the principles outlined in the Basel Declaration, which emphasizes the ethical treatment of animals and the responsible use of animals in scientific research. By following these guidelines, we aimed to ensure the well-being of the animals and to uphold the highest standards of scientific integrity. We would like to express our commitment to the ethical conduct of animal research and extend our appreciation to the relevant regulatory bodies and IACUC for their guidance and oversight throughout the study.

### Animals and experimental design

This study was conducted on Sus scrofa Linnaeus (Yorkshire x Duroc cross), sourced from the Swine Research and Teaching Center at Michigan State University, East Lansing, MI, USA. Piglets underwent either early weaning (EW) at 15 days or later weaning (LW) at 28 days of age. A total of 105 pigs were utilized in this study: 20 early-weaned females (EW-F), 24 later-weaned females (LW-F), 12 early-weaned intact males (EW-IM), 16 later-weaned intact males (LW-IM), 17 early-weaned castrated males (EW-CM), and 16 later-weaned castrated males (LW-CM). Piglets originated from 26 different litters from 26 sows between parity 3–5. Following weaning, piglets were transported to an environmentally controlled nursery unit located approximately 1 mile from the sow farm. Weaned piglets were housed in stainless steel metabolism crates on tenderfoot flooring with four to five pigs/crate. Each crate was equipped with two feeders and adjustable water nipples and pigs were given ad libitum access to feed and water throughout the experiment. The pigs were fed corn-soy based diets in a phase feeding program to meet or exceed the nutrient requirements at each body stage. These rooms housing the crates were environmentally controlled to maintain the thermoneutral environment of all pigs dependent upon age and body weight. Room temperature and humidity were recorded twice daily throughout the study and the room lighting was on a 12:12 h light–dark cycle. Pigs were monitored at least twice daily throughout the study assessing physical appearance, activity, alertness, body condition, and behavior. The pig's environment within the pen and room were also monitored at the same time.

### Vaccination protocol and measurement of vaccine-specific IgG titers

At 6 weeks of age, all pigs were vaccinated with an inactivated porcine circovirus type 2 (PCV2) vaccine (Ingelvac CircoFLEX, Boehringer Ingelheim**)** stored and used according to the manufacturer's guidelines. Immediately prior to vaccination and post-vaccination, blood samples were collected weekly for four weeks using red-top tubes. These tubes were left at room temperature for approximately 30 min to allow the blood to clot, followed by centrifugation to separate the serum. The collected serum was utilized to measure the levels of vaccine-specific PCV2 IgG antibodies, which served as a marker for evaluating the pigs' systemic immune response to the vaccine. The serum IgG titers were assessed at the Veterinary Diagnostic Laboratory at Iowa State University, providing critical insights into the immune functionality and the ability of the pigs to respond to the PCV2 vaccine.

### LPS challenge

**Figure 1:**
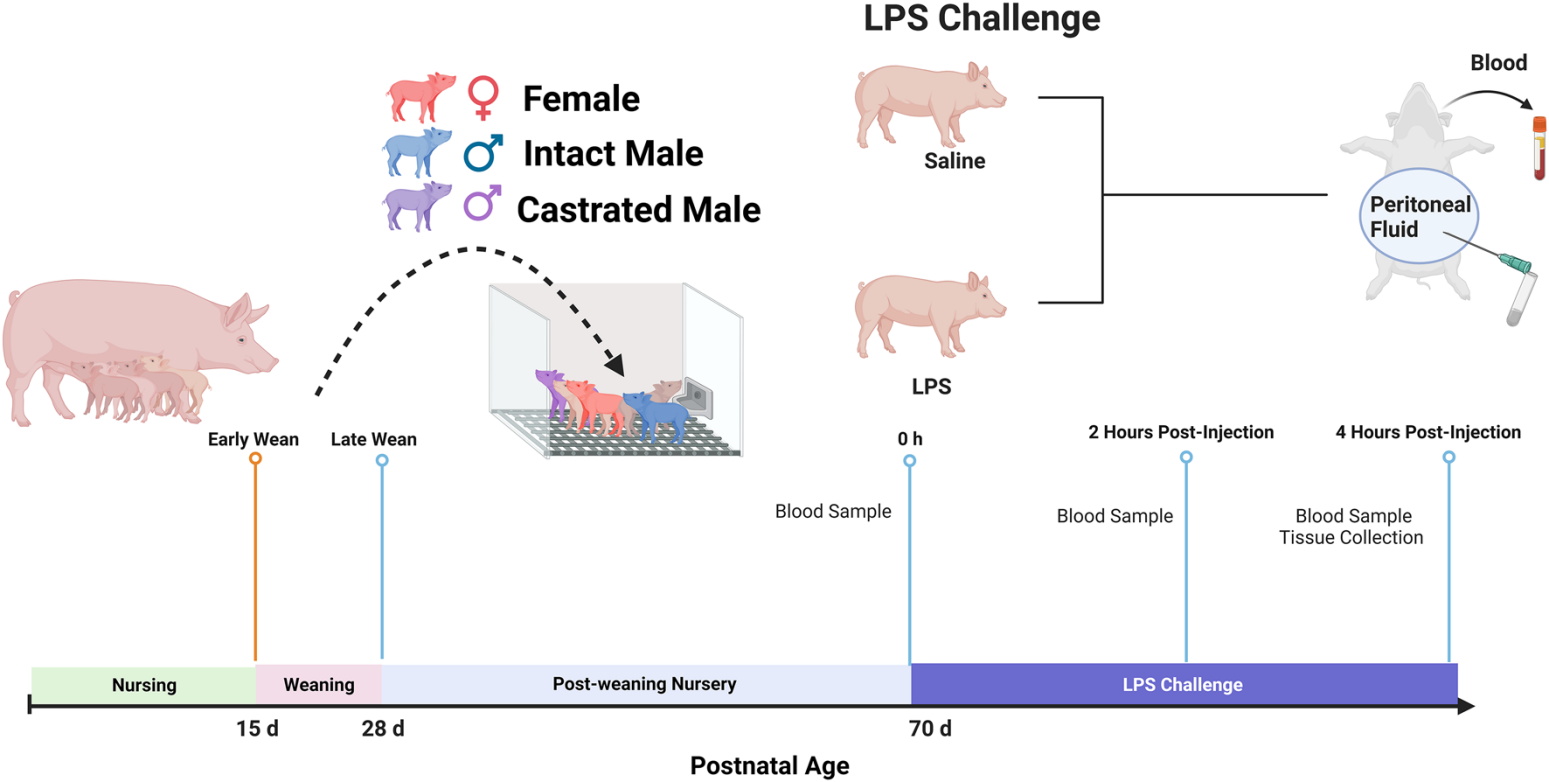
Experimental design. Piglets were weaned from their dam either at 15 days of age (early weaned, EW) or at 28 days of age (later-weaned, LW). Three independent experimental cohorts were conducted. At approximately 70 days of age, pigs within each wean age/sex were randomly assigned to receive via intramuscular injection either LPS (25 μg/kg) or saline. Systemic inflammatory responses were measured via blood sample collection and rectal temperature measurements at 0 h, 2 h, and 4 h post challenge. Animals were euthanized at 4 h and peritoneal fluid and organs were harvested. Created with BioRender.com.

At 10–11-weeks of age, piglets within each weaning age group and sex were randomly assigned to a control group (sterile saline injection) or LPS challenge group. LPS challenge was via an intramuscular injection of 25 µg/Kg LPS (*E. coli* O55:B5, Sigma, Catalog # L2880). Blood samples were collected and rectal temperatures were measured at three time points: 0 h, 2 h, and 4 h post-challenge (Fig. 1) and tissue collection occurred at 4 h post-LPS challenge.

### Blood CBC, immune, and hormonal analysis

Venous blood samples were drawn into K_3_-EDTA tubes and sent to the Pathology Lab at the College of Veterinary Medicine at Michigan State University. Here, they were analyzed for complete blood count (CBC) using an automated hematology analyzer (Advia 2120i, Siemens). All blood smears were subjected to a manual review by a medical technologist to assess red blood cell, platelet, and leukocyte morphology, including assessment for toxic change or band neutrophils. The data from the CBC analysis included counts and percentages of various blood cell types such as red blood cells, white blood cells, platelets, neutrophils, lymphocytes, monocytes, eosinophils, and basophils. Plasma immune and hormonal markers: Measurements of cortisol, testosterone, and cytokines in plasma samples (collected via EDTA tubes) were determined using the enzyme-linked immunosorbent assay (ELISA) kits (TNF-a: R&D Systems Cat. # PTA00, cortisol: Enzo Life Sciences Cat. # ADI-900-071, testosterone: Enzo Life Sciences Cat. # ADI-900-065, CCL2: RayBiotech, Inc. Cat. # ELP-CCL2, insulin: Mercodia Cat#10-1200-01), Luminex assays (TNF, IL6, IL1B, GMCSF and IL2: Millipore Sigma, customized MILLIPLEX MAP porcine cytokine/chemokine Magnetic bead panel, Cat#: PCYTMAG-23K), and colorimetric assay kits (glucose: Abcam Cat# ab65333, NEFAs: Fujifilm Cat# WAKO NEFA HR (2)) following the manufacturer’s instructions.

### Peritoneal fluid collection and processing, and tissue collection

To minimize stress and discomfort for the peritoneal fluid collection, pigs were sedated with a combination of Telazol (2.5 mg/kg), Ketamine (11 mg/kg), and Xylazine (1.5 mg/kg) at a dose of 0.03 mL/kg body weight via intramuscular injection. Once the pigs were fully sedated, the umbilical region of the pigs was thoroughly cleaned with surgical scrub to maintain aseptic conditions. A 16-gauge needle was carefully inserted into the abdomen at the umbilicus to collect the fluid, ensuring that this procedure was performed before any further manipulation of the animal or tissue collection. The pigs were unresponsive to the needle insertion confirming an adequate level of sedation for the procedure. All collected peritoneal fluid samples were then processed within a 3-h window from the time of collection. Initially, the samples were centrifuged at 1200 rpm for 10 min at room temperature. The supernatant was separated and stored in 1.7 microcentrifuge tubes at −80 °C for subsequent cytokine level analysis. The remaining pellet was treated with up to 10 ml of 1× RBC Lysis Buffer (Invitrogen Thermo Fisher Scientific Cat. # 00-4300) and incubated for 5 min at room temperature. Following incubation, the samples underwent a second round of centrifugation under the same conditions. The supernatant was discarded after this step, and the pellet was resuspended in 5 ml of sterile PBS. A portion of this resuspended sample (20 µl) was combined with 20 µl of Trypan Blue for cell count using an automatic cell counter. The cell concentration was adjusted to 75,000 cells per milliliter by adding sterile PBS, post centrifugation and supernatant removal. A 300-µl aliquot of these cell suspensions was used to prepare slides using cytospin. These slides were then stained with the Differential Quik Stain kit (modified Giemsa Cat. # 26096-50) for subsequent analysis. A manual white blood cell differential post- saline or LPS treatment was performed on peritoneal fluid by a boarded clinical pathologist (K.S.) blinded to experimental treatments.

### Euthanasia

Following sedation and peritoneal sample collection described above, the euthanasia procedure was carried out using sodium pentobarbital at a dose of 85.9 mg/kg body weight administered through a catheterized ear vein. The euthanasia procedures were conducted in accordance with IACUC and ARRIVE guideline under an approved animal uses protocol mentioned previously. The use of appropriate sedatives and euthanasia agents, along with proper administration techniques, ensured that the animals were euthanized in a humane and controlled manner. In addition to the peritoneal fluid, additional tissue samples including intestine, lung, liver, and adipose tissue, were harvested immediately after euthanasia for subsequent study.

### Spleen measurements

The spleen's weight and length were recorded and then standardized based on the body weight of each individual pig to account for size variations across the study cohort.

### Anogenital distance

The Anogenital Distance (AGD) was assessed post-euthanasia. AGD refers to the distance from the center of the anus to the base of the genitals. This measurement was taken using a caliper and was performed consistently and carefully to ensure accurate readings. AGD is widely used as a marker of early life androgen exposure with increased prenatal androgen levels, such as in males, resulting in a longer AGD. Therefore, variations in AGD can provide insights into potential disruptions in hormonal activity.

### Statistical analysis

The analysis of repeated measure variables utilized a mixed-effect model in JMP (Version 15, SAS Institute Inc.). This model incorporated the random effect of the pig and the fixed effect of the treatment (either Saline or LPS), sex (CM, F, IM), wean age, and collection time, including their interactions. We considered the collection time as a repeated measure. Post hoc comparisons were undertaken using the Tukey’s adjustments test. Results of this model are presented in terms of significant effects and interactions as *P*-values alongside degrees of freedom and *F-*statistics. For baseline comparisons of repeated measure variables, the data for the 0 h marker was compiled for both saline and LPS treatments across each wean age and biological sex group. This aggregation aimed to scrutinize the impact of these variables before any challenge or isolation. In order to determine specific effects of experimental factors in this study, planned comparisons were made between sexes and between wean age groups within sexes in LPS-treated pigs. Repeated-measures mixed effects models in Prism (Version 10.0.2, Graphpad, Boston, MA, USA) with Geisser–Greenhouse correction were followed by Bonferroni tests. Unless explicitly mentioned, outliers exhibiting significance were excluded. The threshold for statistical significance was a *P*-value of ≤ 0.05, and a *P*-value ≥ 0.05 and ≤ 0.10 indicated a tendency. All data, presented as mean ± SE for each treatment group, excluded non-significant results from the graphs.

## Results

### Impact of sex and wean age on body weight and LPS-induced changes in rectal temperature and spleen weight

**Figure 2:**
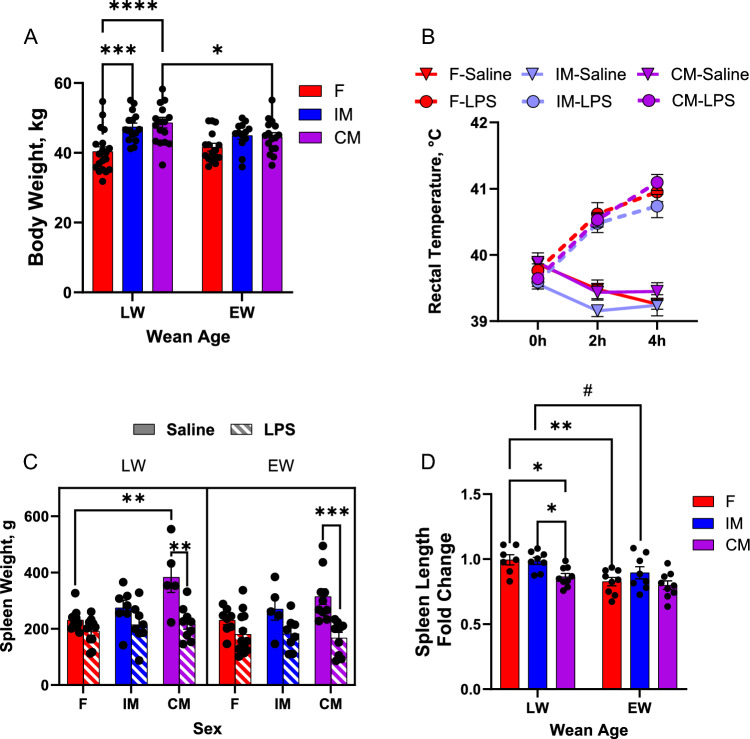
Impact of wean age and sex on body weight and LPS-induced changes in body temperature and spleen size. Piglets were challenged with either LPS or saline at 10–11 weeks of age. Body weight was measured before LPS challenge, rectal temperature was measured at 0 h, 2 h, and 4 h post-challenge, and spleens were collected and measured post-euthanasia. N = 5–13 pigs per experimental group, consisting of early-weaned (EW) and later-weaned (LW) female (F), intact male (IM), and castrated male (CM) pigs. Data are presented as means ± SE. ^#^*P* < 0.1, *P < 0.05, **P < 0.01, ***P < 0.001, ****P < 0.001 Statistics obtained via two-way ANOVA for body weight and spleen length fold change, mixed-effects model with Geisser–Greenhouse correction for rectal temperature, and three-way ANOVA for spleen length. Bonferroni correction applied to planned comparisons. (**A**) Body weight. (**B**) Rectal temperature. (**C**) Spleen weight. (**D**) Spleen length fold change.

Sex had a significant impact on final body weight of pigs (*F*_2,90_ = 12.6,* P* < 0.0001), while trends for wean age (*F*_1,90_ = 2.76,* P* = 0.09) and sex * wean age interactions (*F*_2,90_ = 2.41,* P* = 0.10) were observed (Fig. 2A). Bonferroni planned comparison tests showed LW-IM and -CM had greater (*P* < 0.001) final body weights compared with LW-F, but there were no significant sex differences among EW pigs, due to lower body weights in EW-MI and EW-MC pigs. Specifically, wean age had a significant impact on final body weight in MC pigs with EW-CM having lower final body weights compared with LW-CM (*P* < 0.05). However, EW did not have a significant impact on final body weight in F or IM pigs. (Fig. 2A).

An LPS * time interaction (*F*_1,71.9_ = 230,* P* < 0.0001) for body temperature was observed with pigs from all experimental groups exhibiting elevated rectal temperatures at 2 h and 4 h post-LPS challenge compared with saline controls (Fig. 2B). There was a trend for a main effect of sex (*F*_2,76.5_ = 2.66,* P* = 0.076) and EW-F had higher temperature than LW-F when measured at baseline (*P* < 0.05). There was no effect of wean age (*F*_1,77_ = 0.01,* P* = 0.918) or wean * sex interactions, (*F*_2, 76.5_ = 0.16,* P* = 0.845).

Splenic contraction, a physiological response to sepsis and stress mediated by sympathetic nervous system activation resulting in release red blood cells (RBCs) and immune cells from the spleen into the circulation, was assessed as an indirect gross index of neurohormonal response to LPS challenge by measuring spleen weight and length in pigs in saline and LPS challenged groups at 4 h post LPS challenge. At basal conditions (Saline-treated controls) LW-CM pigs had greater spleen weights compared with LW-F pigs (*P* < 0.05). LPS challenge induced some degree of splenic contraction, measured as reductions in spleen weight (Fig. 2C) and length (Fig. 2D) 4 h post-LPS challenge, in pigs from all treatment groups. However, the degree of splenic contraction was influenced by biological sex and wean age. Among LW pigs, CM pigs had greater splenic contraction responses than F and IM (*P* < 0.05). However, this difference was not observed in EW-MC pigs. EW-F pigs exhibited significantly greater splenic contraction than LW-F (*P* = 0.001) and a tendency (*P* = 0.069) for enhanced splenic contraction in EW-MI was also observed in IM pigs.

### Effects of wean age and sex on CBC parameters in LPS-challenged pigs.

Effects of weaning age, sex, and LPS treatment on various CBC parameters are summarized in Supplemental Table 1 with corresponding statistical values summarized in Supplemental Table 2.

#### Segmented neutrophils

**Figure 3:**
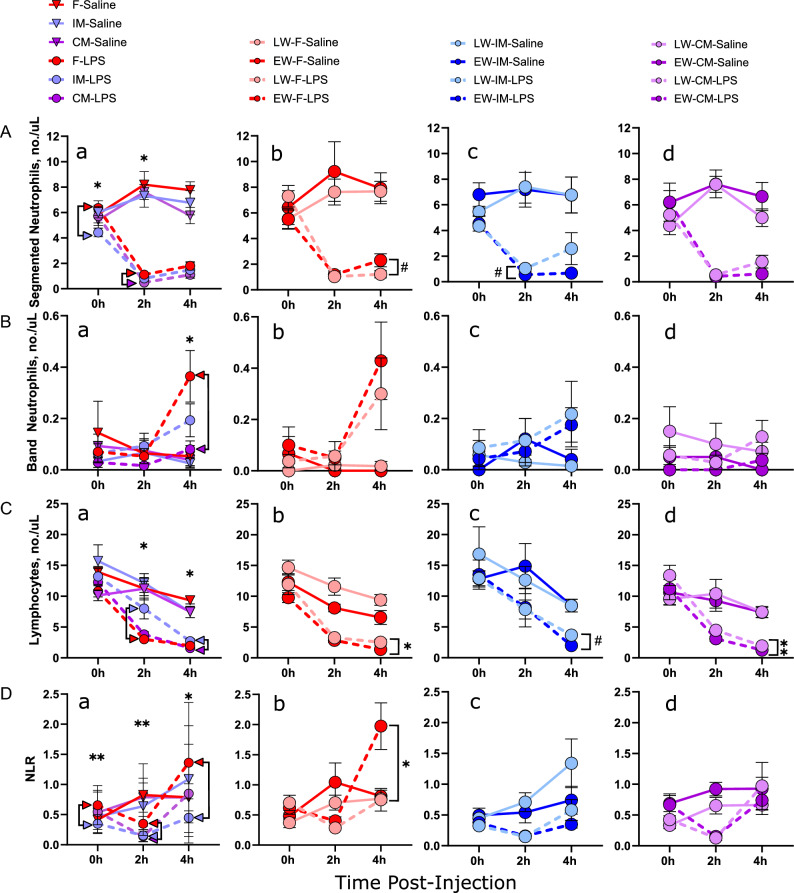
Effect of wean age and sex on CBC parameters in response to LPS challenge over 4 h. Piglets were challenged with either LPS or saline at 10–11 weeks of age, and venous blood samples were taken at 0 h, 2 h, and 4 h post-challenge and analyzed for complete blood count (CBC). N = 5–11 pigs per experimental group, consisting of early-weaned (EW) and later-weaned (LW) female (F), intact male (IM), and castrated male (CM) pigs. Data are presented as means ± SE. Statistics obtained via repeated-measures mixed-effects model with Bonferroni correction applied to planned comparisons. ^#^*P* < 0.1, *P < 0.05, **P < 0.01. Subfigure (**a**) in each row: sex differences between LPS-treated groups. Colored arrowheads specify significant differences. Subfigures (**b**–**d**): wean age differences within LPS-treated groups. Brackets indicate comparisons between LPS-treated groups. Saline-treated groups are included for illustrative purposes. (**A**) Segmented neutrophils, (**B**) band neutrophils, (**C**) lymphocytes. (**D**) neutrophil-to-lymphocyte ratio (NLR).

Significant main effects were noted for time (*F*_1,74.1_ = 94.4,* P* < 0.0001), LPS (*F*_1,78.6_ = 70.0,* P* < 0.0001) and time * LPS (*F*_1,74.1_ = 131,* P* < 0.0001) with LPS challenge inducing a marked reduction in neutrophil numbers at 2 and 4 h (*P* < 0.001) post-LPS challenge (Fig. 3A, Supplemental Table 1). There were no main effects of weaning age (*F*_1, 78.6_ = 0.120,* P* = 0.730), but a trend for a sex effect was observed (*F*_2,78.6_ = 3.00,* P* = 0.056) with F pigs exhibiting significantly higher neutrophil counts at 2 h post LPS, compared with CM pigs (*P* = 0.014). A weaning age * LPS interaction (*F*_1.78.6_ = 4.41,* P* = 0.039) and a 4-way interaction involving sex, weaning age, time, and treatment (*F*_2, 74.1_ = 3.78,* P* = 0.028) were found.

#### Band neutrophils

Analysis of band neutrophils (Fig. 3B), which are immature neutrophils that increase in the circulation in response to an overwhelming inflammatory response, revealed a significant time * LPS effect (*F*_1,77.1_ = 13.4,* P* = 0.0005) with increased band neutrophils observed at 4 h post-LPS challenge. Comparisons between the sexes indicated that F pigs exhibited greater band neutrophil responses compared with CM at the 4 h time point (*P* = 0.048). While weaning age alone had no significant effect on band neutrophil numbers (*F*_1,80.9_ = 0.134,* P* = 0.715) a sex * weaning age interaction (*F*_2,81_ = 3.25,* P* = 0.044) was found. Specifically, in LW-F only, a significant increase in band neutrophil counts was observed from 2 to 4 h (*P* = *0.0*38). Together, these data highlight sex differences in neutrophil responses, with females displaying a greater increase in band neutrophils.

#### Lymphocytes

Lymphocyte numbers (Fig. 3C) declined within 2 h post-LPS challenge in all experimental groups (time * LPS interaction; (*F*_1,78.3_ = 245,* P* < 0.0001)). A significant three-way interaction was observed between sex, LPS, and time (*F*_2,78.2_ = 3.39* P* = 0.0388) and between wean age, LPS, and time (*F*_2,78.3_ = 19.7,* P* > 0.0001). With regards to sex differences, IM exhibited a less pronounced initial decline in lymphocyte numbers measured at 2 h post-LPS challenge, compared with F and CM; however, lymphocyte number continued to decline IM and were similar to F and CM by 4 h post-LPS. We observed a main effect related to weaning age, where EW pigs showed larger decreases in lymphocyte numbers compared to LW pigs. This effect was statistically significant in both the F group (P = 0.047) and the CM group (P = 0.008). In the IM group, the effect was nearly significant but slightly above the threshold for statistical significance (P = 0.053).

#### NLR

No significant main effects of sex (*F*_2,79_ = 2.73,* P* = 0.071), weaning age (*F*_1,79_ = 0.678,* P* = 0.413), or treatment (*F*_1,79_ = 0.095,* P* = 0.076) were observed for NLR. Significant three-way interactions were observed between sex, weaning age, and time (*F*_2,74.4_ = 5.72,* P* = 0.0049), weaning age, time, and treatment (*F*_1,74.4_ = 4.16,* P* = 0.0451), and a significant four-way interaction was found between sex, weaning age, time, and treatment (*F*_2,74.4_ = 5.12,* P* = 0.0082). The NLR was significantly reduced from 0 to 2 h in LPS-treated F, IM, and CM pigs (*P* < 0.01–001), then significantly increased in all LPS-treated groups from 2 to 4 h (*P* < 0.01–*P* < 0.0001) (Fig. 3D). The greatest increase was observed in F-LPS, with the NLR at 4 h being significantly higher than that of IM-LPS (*P* = 0.015). The most notable difference in NLR between wean age groups was observed in female pigs, with EW-F exhibiting a fourfold greater increase in NLR compared with LW-F measured at 4 h post-LPS (*P* = 0.02). This increase appeared to be largely driven by the increase in neutrophil numbers in EW-F.

#### WBC

Time (*F*_1,82_ = 119.8,* P* < 0.001) and LPS treatment (*F*_1,83.4_ = 30.8,* P* < 0.0001) had significant effects on white blood cell (WBC) count, with a significant interaction between these factors (*F*_1,82_ = 24.3,* P* < 0.0001). At 2-h and 4-h timepoints, all LPS-treated pigs exhibited significantly reduced WBC levels (*P* < 0.05-p < 0.0001). Although no main effects of sex (*F*_2,83.3_ = 2.05,* P* = 0.136), weaning age (*F*_1,83.4_ = 1.47,* P* = 0.228), or sex * weaning age interaction (*F*_2,83.3_ = 0.002,* P* = 0.998) were observed, planned comparisons revealed some differences. At 2 h post-LPS challenge, IM-LPS had higher WBC counts than F-LPS (*P* = 0.042) and tended to have higher than CM (*P* = 0.051) Additionally, EW-CM tended to have higher WBC numbers compared to LW-CM at the 4-h post-LPS challenge (*P* = 0.083).

#### Monocytes

Significant effects of time (*F*_1,80.8_ = 56.8,* P* < 0.0001) and LPS treatment (*F*_1,81.9_ = 8.31,* P* = 0.005) were observed, along with a significant interaction between time and LPS treatment (*F*_1,80.8_ = 7.05,* P* = 0.010). There was a significant main effect of sex (*F*_2,81.8_ = 3.26,* P* = 0.044). This effect was not significant in planned comparisons at specific timepoints, but EW-F tended to exhibit higher monocyte levels than LW-F at 4 h post-LPS challenge (*P* = 0.067). The interaction between sex and weaning age was not significant (*F*_2,81.8_ = 1.30,* P* = 0.278), but a significant four-way interaction between sex, weaning age, time, and LPS treatment (*F*_2,80.7_ = 4.41,* P* = 0.015) was observed.

#### Eosinophils

Wean age (*F*_1,80.6_ = 9.17,* P* = 0.015) and time (*F*_1,80.2_ = 82.8,* P* < 0.0001), along with a time * weaning age interaction (*F*_1,80.2_ = 7.84,* P* = 0.006), had significant effects on eosinophil numbers. LPS challenge induced reductions in eosinophil numbers by 2 h post-LPS challenge in all groups except for EW-IM and EW-CM pigs (*P* < 0.05–*P* < 0.001). EW-IM also had reduced eosinophil numbers compared to LW-IM pigs (*P* < 0.01) at baseline (0 h), indicating an effect of wean age in intact males.

#### Basophils

Significant main effects of sex (*F*_2,81_ = 5.54,* P* = 0.006), time (*F*_1,78.6_ = 10.6,* P* = 0.002), and LPS treatment (*F*_1,80.9_ = 5.83,* P* = 0.018) were detected. Significant sex * LPS interactions (*F*_2,81_ = 4.18,* P* = 0.019) and weaning age * LPS treatment interactions (*F*_1,80.9_ = 4.21,* P* = 0.044) were also observed.

#### Red blood cells (RBCs)

Significant effects of time (*F*_1,81_ = 12.2,* P* < 0.001) and LPS treatment (*F*_1,83.3_ = 26.5,* P* < 0.001), along with a time * LPS treatment interaction (*F*_1,81.1_ = 47.5,* P* < 0.001), were identified for RBC numbers. A three-way interaction between sex * wean age * LPS was observed (*F*_2,83.8_ = 3.24,* P* = 0.044). Specifically, there was a significant increase in RBC levels in response to LPS challenge for LW-CM (*P* = 0.049, 0 h vs 2 h) and EW-CM (*P* = 0.048, 0 h vs 2 h).

#### Hematocrit (Hct), hemoglobin (Hgb) levels and platelets

Significant effects of sex, time, and LPS treatment were observed, with interactions between time and LPS treatment leading to increased levels post-challenge. Weaning age showed a significant interaction with sex and LPS treatment for both Hct and Hgb levels. Platelet levels were influenced by time and LPS treatment, with reductions post-LPS challenge in all pigs. Interactions between time, sex, and LPS treatment were significant, and weaning age showed a significant interaction with LPS treatment.

### Plasma chemokine and cytokine concentrations

#### TNF-α

**Figure 4:**
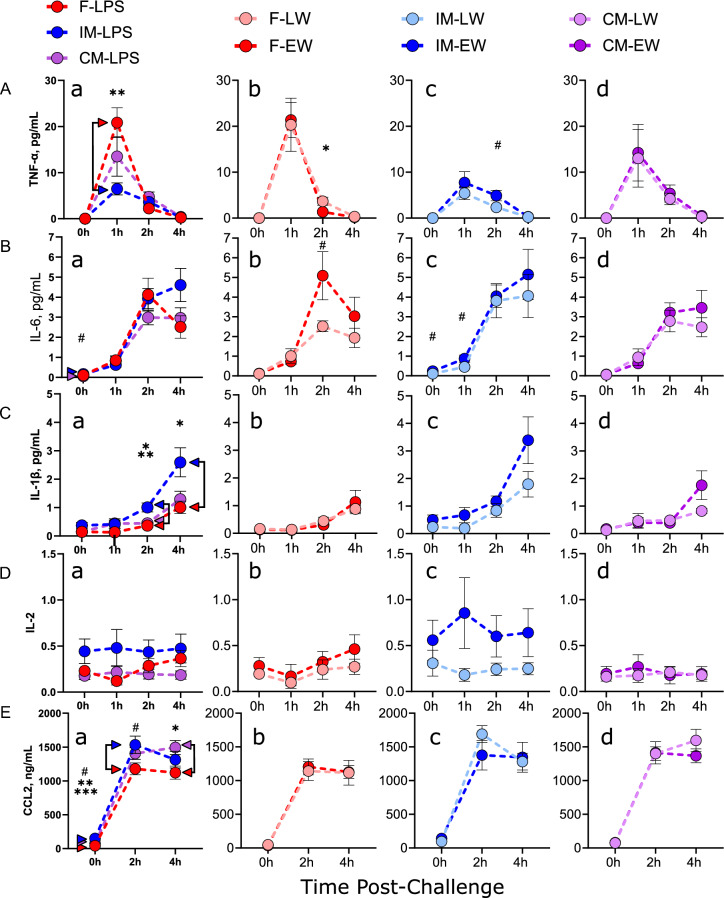
Effects of wean age and sex on blood cytokine and chemokine levels in LPS-challenged pigs. Cytokine and chemokine measurements were determined using ELISA kits and plasma from venous pig blood collected at 0 h, 1 h, 2 h, and 4 h post-LPS challenge. N = 8–15 per experimental group, consisting of early-weaned (EW) and later-weaned (LW) female (F), intact male (IM), and castrated male (CM) pigs. Data are presented as means ± SE. ^#^*P* < 0.1, *P < 0.05, **P < 0.01, ***P < 0.001. Statistics obtained via repeated-measures mixed-effects model with Bonferroni correction applied to planned comparisons. Subfigure (**a**): effects of sex; subfigures (**b–d**): wean age effects within sexes. (**A**) TNF-α concentrations. (**B**) IL-6 concentrations. (**C**) IL-1β concentrations. (**D**) IL-2 concentrations. (**E**) CCL2 concentrations.

Plasma TNF-α concentrations increased in response to LPS challenge across all experimental groups, reaching peak concentrations at 1 h post LPS injection and returning to baseline levels by 4 h (Fig. 4A). At 1 h post-challenge, there was a significant effect of sex (*F*_2,23_ = 4.57,* P* = 0.02). Beyond the 1-h time point, there were no significant effects observed for sex, weaning age, or sex * weaning age interactions. However, at 2 h post LPS challenge, a trend for a sex * wean age interaction (*F*_2,39_ = 2.66,* P* = 0.08) was observed. Planned comparisons at this time point indicated that F pigs exhibited significantly higher TNF-α levels compared to IM pigs (*P* < 0.05).

#### IL-6

A significant effect of sex was observed at the 0 h timepoint (*F*_2,30_ = 5.16,* P* = 0.012) with IM pigs tending to have greater IL-6 levels compared with CM pigs (*P* = 0.07). Plasma IL-6 concentrations were increased in response to LPS-challenge by 1 h post-LPS (Fig. 4B). The planned comparison of wean age groups within sexes revealed a significant time * wean age interaction in F only (*F*_3,23_, = 3.79,* P* = 0.024) as well as a trend for higher IL-6 in EW-F at 2 h (*P* = 0.068), indicating that EW in F pigs may exhibit and enhanced IL-6 responses.

#### IL-1β

LPS challenge increased IL-1β levels at 2 h and 4 h in all experimental groups, and levels were affected by sex and weaning age (Fig. 4C). At 4 h, both sex (*F*_2,45_ = 6.07,* P* = 0.005) and weaning age (*F*_1,42_ = 5.48,* P* = 0.024) had significant main effects on IL-1β levels. At 2 h post-challenge, IM had higher IL-1β levels than F (*P* = 0.005) and CM (*P* = 0.011). At 4 h, IM had significantly higher levels than F (*P* = 0.029). There was no significant difference between F and CM at any time point. These results suggest that IM had greatest IL-1β responses to LPS and that this was attributed to gonadal status.

#### IL-2

There were no overall effects of sex, weaning age, or LPS-challenge on plasma IL-2 concentration. Planned comparisons revealed that in F pigs only, IL-2 concentrations decreased from 0 to 1 h, then significantly increased over the 4 h LPS challenge period (*P* < . 0001)(Fig. 4D).

#### CCL2

CCL2 levels were influenced by sex and LPS challenge (Fig. 4E). At baseline (0 h), IM and CM had higher CCL2 levels than F pigs (*P* < 0.01) and IM tended to have higher than CM (*P* = 0.079). Wean age and the interaction between sex and weaning age were not significant factors. LPS challenge increased CCL2 levels in all pigs at 2 h and 4 h, but there were no significant overall effects of sex, weaning age, or their interaction. These results indicate that males (IM and CM) have a higher basal CCL2 level than females, but that LPS challenge induces a similar increase in CCL2 in all pigs regardless of sex or weaning age.

#### Peritoneal fluid immune cell analysis

**Figure 5:**
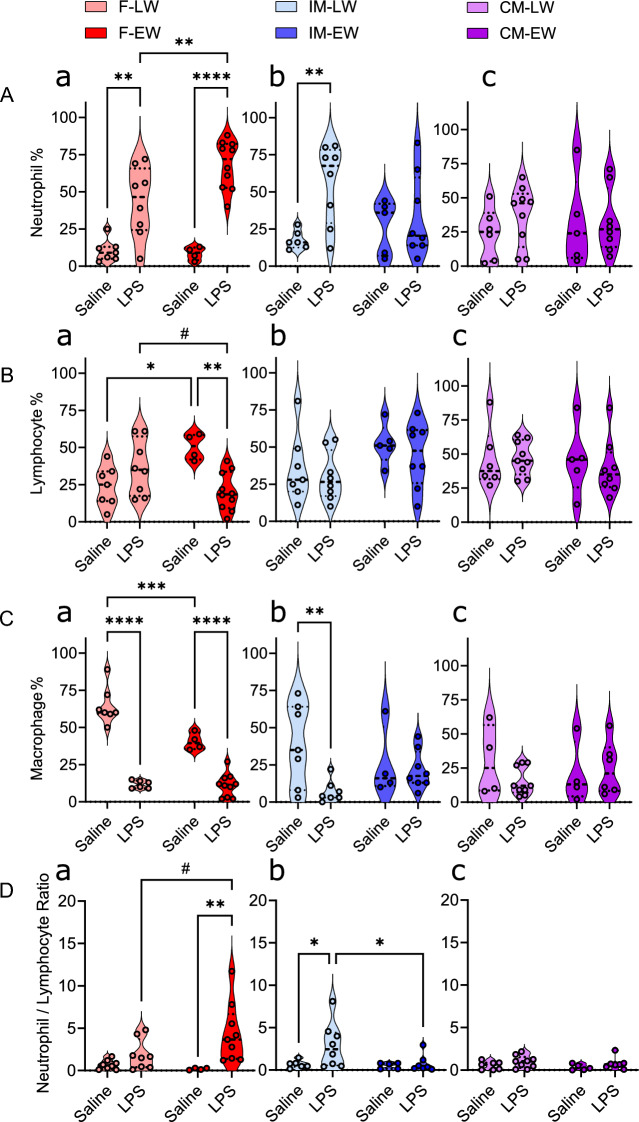
Wean age and sex effects on LPS-associated peritoneal immune cells. Peritoneal fluid was collected under sedation at 4 h post-challenge and processed within 3 h of collection. Samples were evaluated by a clinical pathologist blinded to experimental treatments. Data are presented as means ± SE with n = 5–10 animals/experimental group, consisting of early-weaned (EW) and later-weaned (LW) female (F), intact male (IM), and castrated male (CM) pigs injected with either LPS or saline (SAL). *P < 0.05, **P < 0.01, ***P < 0.001, ****P < 0.001. Statistics obtained via two-way ANOVA with Bonferroni correction applied to planned comparisons. (**A**) Neutrophil percentages. (**B**) Lymphocyte percentages. (**C**) Macrophage percentages. (**D**) Neutrophil-to-lymphocyte ratio (NLR).

Intramuscular administration of LPS activates the innate immune system through recognition by Toll-like receptor 4 (TLR4). This leads to the release of pro-inflammatory mediators that increase vascular permeability, facilitating the mobilization of immune cells, including neutrophils, into the bloodstream. As these immune factors circulate, they promote neutrophil attachment and migration to various tissues, including the peritoneum. This process exemplifies how an intramuscular injection of LPS can result in a localized immune response within the peritoneum, marked by the recruitment of neutrophils. We analyzed systematic immune response to LPS challenge by measuring the changes in WBC composition in peritoneal fluid (PF) (Fig. 5).

#### PF neutrophils

Neutrophils are key cells that migrate from the bloodstream to tissue sites of infection or inflammation. LPS challenge had a significant effect on neutrophil percentages (Fig. 5) in female (*F*_1,25_ = 52.18,* P* < 0.0001) and intact male pigs (*F*_1,23_ = 6.2,* P* = 0.02). The impact of LPS on neutrophil percentages was modulated by sex and weaning age. In females, EW-F had a greater PF neutrophil response than LW-F, indicating a heightened neutrophil migration in response to LPS because of EW. In contrast, EW-IM responded oppositely than females by exhibiting a suppressed PF neutrophil response compared with LW-IM, suggesting that early weaning may impair the PF neutrophil migration in IM. Castrates had lower PF neutrophil responses to LPS compared with IM regardless of weaning age, implying that castration may also compromise the neutrophil migration.

#### PF lymphocyte

The F-EW-LPS group exhibited a significant (*P* = 0.003) reduction in lymphocyte percentages (mean = 20.30%, SEM = 4.07) compared with saline-treated F-EW pigs (mean = 50.50%, SEM = 4.42). This significance was not observed in LW-F pigs. There were no significant effects of LPS on PF lymphocyte percentages in IM or CM pigs. The corresponding neutrophil to lymphocyte ratio (NLR) showed that F-EW-LPS pigs exhibited the greatest increase in PF NLR (mean = 4.399, SEM = 1.168) compared with saline-treated F-EW (mean = 0.183, SEM = 0.057), signifying an intense immune response to LPS.

#### PF macrophage

PF macrophage percentages were affected by sex and LPS challenge, with LW-F pigs having the highest concentrations. LPS challenge significantly decreased PF macrophage percentages in F pigs, (*F*1,64 = 32.1,* P* < 0.0001), especially in EW-F, which had the lowest PF macrophage percentages after LPS challenge. LPS challenge also had a significant effect on IM pigs (*F*_1,22_ = 5.5,* P* = 0.028). The percentage of PF macrophages was not altered in LPS-challenged CM pigs. These results indicated that early weaning and LPS challenge induce PF macrophage depletion in females and intact males. A decrease in PF macrophage percentages may indicate a severe inflammatory response that leads to PF macrophage death or pyroptosis, a form of inflammatory cell death that releases pro-inflammatory cytokines and damage-associated molecular patterns, or their migration to other sites such as regional lymph nodes or injured tissues^16^.

Together, these findings underscore the differential effects of early weaning across sexes and castration status. Female pigs that were EW manifested an exacerbated local inflammatory response characterized by enhanced neutrophil recruitment, significant increase in NLR (Fig. 5D), and a marked depletion of macrophages. These results establish that females are distinctly sensitive to EW, demonstrating a pronounced inflammatory response that may potentially contribute to increased susceptibility to inflammatory conditions.

On the contrary, IM pigs exhibited a suppressed response, with EW resulting in impaired neutrophil migration, highlighting a potential vulnerability in these animals' inflammatory response. CM pigs, however, displayed a near-absent response with no significant white blood cell differential response to LPS, revealing an apparent impaired response, regardless of wean age.

#### Weaning age and sex effects on vaccine-induced IgG titers

**Figure 6:**
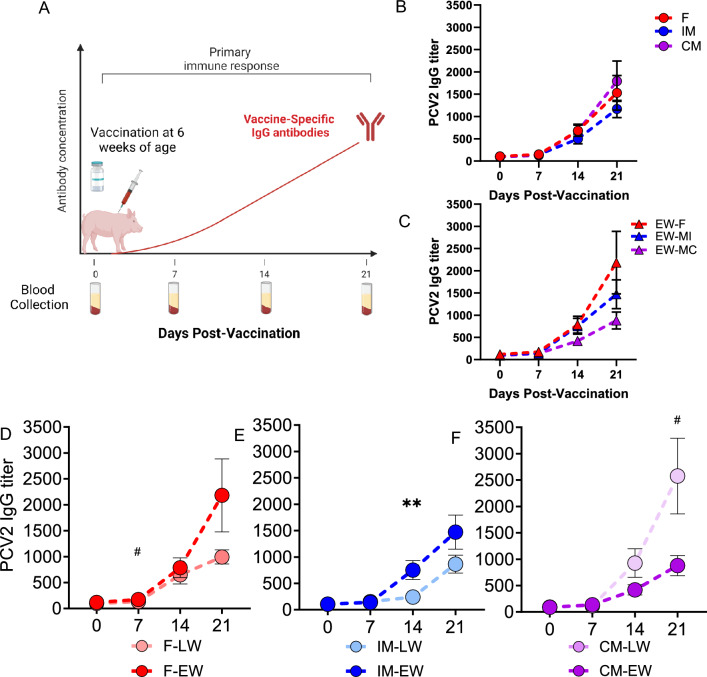
Wean age and sex effects on vaccine-induced IgG responses. Experimental design is outlined in (**A**) (created with BioRender.com). Blood samples were collected at 0, 7, 14, and 21 days post-vaccination with an inactivated porcine circovirus type 2 (*P*CV2) vaccine, and serum PCV2-IgG was quantified to determine the vaccine-specific immune response.  Data are presented as means ± SE. *P < .05, ^#^*P* < 0.1. N = 7–8 per experimental group, consisting of early-weaned (EW) and later-weaned (LW) female (F), intact male (IM), and castrated male (CM) pigs. Data are expressed as main effects of sex (**B**) and sex x EW (**C**). The data were subjected to a natural logarithmic transformation for normality prior to analysis but are presented as original values. A nonlinear regression revealed significant differences between EW and LW titer curves in IM (*P* = 0*.*02; Panel **E**) and CM (*P* = 0*.*01; Panel **F**), while a trend was observed in F (Panel **D**). Timepoint comparison statistics obtained via repeated-measures mixed-effects model with Bonferroni correction for planned comparisons.

To complement the LPS model of innate immune activation, we used vaccination-induced IgG responses as a model of adaptive immune activation as illustrated in Fig. 6A. Specifically, we measured the serum PCV2-IgG titer responses to PCV2 vaccination to obtain an index of adaptive immune response in pigs. Across all sexes (F, IM, and CM) (Fig. 6B) and EW (Fig. 6C) and LW (not shown) groups, there was a pronounced effect of time on the titers (*P* < 0.0001) with increasing titers over the 4-week test period. In F pigs, no significant effects of wean age on IgG titers were observed; however, there was a trend towards higher titers in the EW-F vs LW-F at day 7 (*P* = 0.057) (Fig. 6D). In both IM (Fig. 6E) and CM (Fig. 6F), a nonlinear regression revealed distinct titer curves for EW and LW pigs (*P* = 0.02 and* P* = 0.01, respectively). Additionally, significant interactions were observed between time and wean age in both IM (*F*_3,38_ = 5.9,* P* = 0.002) and CM (*F*_3,34_ = 2.95,* P* = 0.046) groups. Specifically, in the IM group, EW-IM pigs displayed significantly higher titers at day 14 post-vaccination (*P* = 0.004), compared with LW-IM. In contrast, a tendency for lower titers in the EW-CM group was observed at day 21 post-vaccination (*P* = 0.08).

#### Plasma cortisol, testosterone and estradiol

**Figure 7:**
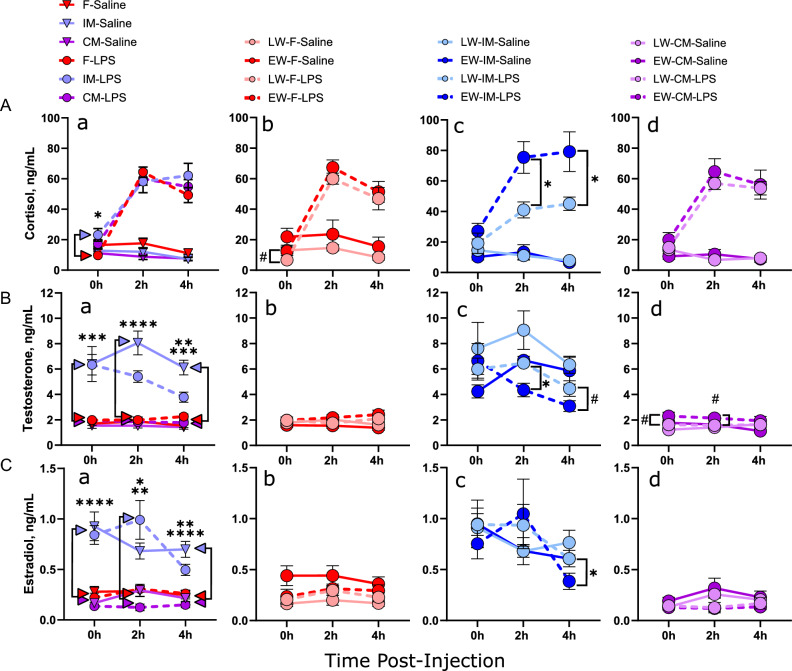
Effects of early weaning, sex, and LPS challenge on plasma levels of cortisol, testosterone, and estradiol. Piglets were challenged with either LPS or saline at 10–11 weeks of age, and venous blood samples were taken at 0 h, 2 h, and 4 h post-challenge and plasma hormone levels were analyzed. N = 5–11 pigs per experimental group, consisting of early-weaned (EW) and later-weaned (LW) female (F), intact male (IM), and castrated male (CM) pigs. Data are presented as means ± SE. ^#^*P* < 0.1, *P < 0.05, **P < 0.01, ***P < 0.001, ****P < 0.001 Statistics obtained via repeated-measures mixed-effects model with Bonferroni correction applied to planned comparisons. ^#^*P* < 0.1, *P < 0.05, **P < 0.01. Subfigure (**a**) in each row: sex differences between LPS-treated groups. Colored arrowheads specify significant differences. Subfigures (**b–d**): wean age differences within LPS-treated groups. Brackets indicate comparisons between LPS-treated groups. Saline-treated groups are included for illustrative purposes. (**A**) Cortisol levels, (**B**) testosterone levels, (**C**) estradiol levels.

This study explored the interplay of weaning age and sex on the modulation of the HPA axis response and sex hormone levels, specifically focusing on plasma cortisol, testosterone, and estradiol levels in pigs subjected to an LPS challenge. Our results highlighted a significant impact of LPS challenge (*F*_1,82_ = 82.1,* P* < 0.0001) and time (*F*_1,81.9_ = 54.2,* P* < 0.0001) on cortisol levels (Fig. 7A). All experimental groups demonstrated an increase in cortisol levels at the 2 and 4-h post-LPS challenge indicating activation of the HPA axis. A main effect of wean age was observed (*F*_1,82_ = 4.34,* P* = 0.040), with EW pigs exhibiting elevated cortisol responses, compared to LW pigs. A three-way interaction among sex, wean age, and LPS challenge (*F*_2,82_ = 3.33,* P* = 0.041) was observed. Specifically, EW-IM exhibited greater LPS-induced plasma cortisol levels than LW-IM at 2 h (*P* = 0.016) and at 4 h (*P* = 0.036) post-LPS challenge.

Given the interplay of HPA axis activity, sex hormones and the immune response, we evaluated the blood levels of testosterone and estradiol. As anticipated, IM pigs consistently showed higher testosterone (Fig. 7B) and estradiol (Fig. 7C) levels compared to F and CM pigs within the same weaning group. The IM pigs in this study were prepubertal, and therefore the elevated testosterone levels reflect the prepubertal androgen surge that begins soon after birth and persists until ~ 4 months of age. The high testosterone levels in IM were found to be influenced by an interaction between wean age and LPS. Following LPS challenge, EW-IM pigs exhibited a significant decline in plasma testosterone levels resulting in significantly lower levels 2 h post-LPS challenge compared with LW-IM pigs (*P* = 0.013) and a trend towards lower levels at 4 h (*P* = 0.087). In contrast to IM, neither LPS nor wean age impacted testosterone levels in CM or F pigs. Significant sex effects on plasma estradiol levels were also observed (*F*_2,78.2_ = 45,* P* < 0.0001) with IM pigs having higher plasma estradiol levels than F and CM pigs. Similar to testosterone, LPS induced a decline in estradiol in EW-IM pigs indicated by significantly lower levels than LW-IM at 4 h post LPS challenge (*P* = 0.041). Examination of anogenital distance (AGD) and gonad weights in intact male (IM) pigs, both of which are androgen-sensitive measurements, indicated reduced testosterone levels in early-weaned (EW-IM) pigs compared to late-weaned (LW-IM) pigs (Supplemental Fig. 1). This finding may suggest that early weaning has a negative impact on the ability of EW-IM pigs to produce and maintain androgen levels during challenges. In summary, these results underscore the significant roles of weaning age and sex on plasma cortisol, testosterone, and estradiol responses during an LPS challenge. Notably, early weaning and IM status both correlate with heightened cortisol responses, and early weaning seems to negatively influence testosterone levels during an LPS challenge.

### LPS challenge and wean age affect metabolic factors related to immune function

Metabolic factors such as insulin, glucose, and NEFA are involved in the regulation of immune function and inflammation and can be influenced by biological sex and early life adversity.

#### Glucose

**Figure 8:**
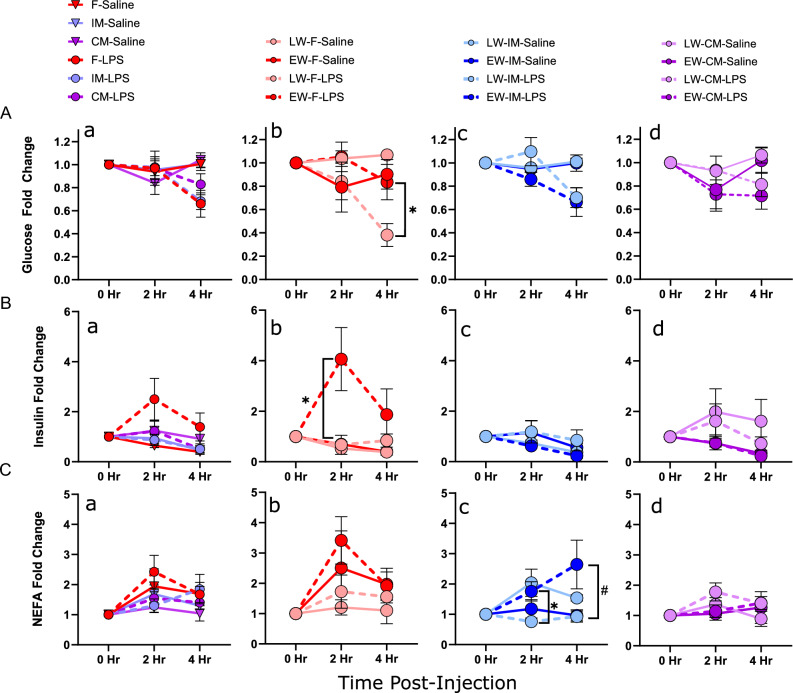
Wean age and sex effects on LPS-induced changes in metabolic parameters. Piglets were challenged with either LPS or saline at 10–11 weeks of age. Venous blood samples were taken at 0 h, 2 h, and 4 h post-challenge and metabolic factor levels were analyzed. N = 5–11 pigs per experimental group, consisting of early-weaned (EW) and later-weaned (LW) female (F), intact male (IM), and castrated male (CM) pigs. Data are presented as means ± SE. ^#^*P* < 0.1, *P < 0.05, **P < 0.01, ***P < 0.001, ****P < 0.001. Statistics obtained via repeated-measures mixed-effects model with Bonferroni correction applied to planned comparisons. ^#^*P* < 0.1, *P < 0.05, **P < 0.01. Subfigure (**a**) in each row: sex differences between LPS-treated groups. Subfigures (**b–d**): wean age differences within LPS-treated groups. Brackets indicate comparisons between LPS-treated groups Saline-treated groups are included for illustrative purposes. (**A**) Glucose fold change. (**B**) Insulin fold change. (**C**) Non-esterified fatty acid (NEFA) fold change.

It is well established that LPS challenge can alter the metabolic status of animals by inducing hyperglycemia, insulin resistance, and lipolysis to mobilize resources to fuel the immune response. In the female groups, the LW-F group treated with LPS showed a ~ 62% decline in blood glucose levels by 4-h post-LPS challenge compared with baseline (0 h) (*P* = 0.01) indicating a marked hypoglycemia (Fig. 8A). Unexpectedly, the hypoglycemia response was blunted in EW-F pigs, indicated by a greater glucose level in EW-F compared with LW-F at 4 h post-LPS challenge (*P* = 0.029). In contrast to EW-F, EW-IM exhibited a more rapid and greater decline in blood glucose levels at 2 h compared with LW-IM pigs. Blood glucose levels continued to decline at 4 h post-LPS challenge, at which time LW- and EW-IM blood glucose levels were similar and ~ 44% lower than baseline. In the CM groups, both the LW-CM and EW-CM exhibited a numerical decline in blood glucose levels post-LPS challenge; however, this was not statistically significant at any time point.

#### Insulin

A significant effect of sex was observed in insulin levels (Fig. 8B) post-LPS challenge (*F*_2,66.8_ = 8.2,* P* < 0.001). At 2-h post-LPS challenge, statistically significant differences were observed in female pigs. EW-F pigs responded to LPS with a significant rise in blood insulin levels that was greater than that of LW-F (*P* = 0.035). By 4 h post-LPS, insulin levels declined in EW-F pigs to levels similar to the IM and CM groups.

#### NEFA

There were significant effects of time (*F*_1,80.7_ = 15.3,* P* = 0.0002) and LPS (*F*_1,81_ = 17.4,* P* = 0.0001) on NEFA levels, as well as a significant interaction between time and treatment (*F*_1,80.7_ = 4.77,* P* = 0.032) (Fig. 8C). A wean age effect was observed in IM pigs, with EW-IM having higher NEFA concentrations than LW-IM (*P* = 0.021) at 2 h and a trend toward the same effect at 4 h (*P* = 0.077). At 4 h, no significant differences were observed between any of the groups.

## Discussion

In this study, we sought to address the major gap in knowledge regarding the impact of weaning age and biological sex on immune responses to later life challenge in pigs. Here we focused primarily on the systemic immune response by evaluating blood cytokine/chemokine levels, CBC parameters, and peritoneal WBC analysis. The immune assays were complemented by hormonal and metabolic profile analysis. Together, results from the current study reveals a complex interplay between weaning age, biological sex/castration, and immune response to LPS challenge, with significant interactions observed in several immune parameters. These results have important implications for understanding the long-lasting effects of early weaning on the immune system and the role of biological sex in modulating immune responses, which can inform management strategies to improve swine health, welfare, and productivity.

Sex had a significant impact on the immune responses measured in this study. While variations in the immune response to LPS were evident across the sexes and dependent upon experimental condition, a consistent pattern of heightened immune responses in females compared with males (IM and CM) was found. Female pigs exhibited greater, band neutrophils, TNF-α and IL6 (trend), and enhanced immune response to early weaning including LPS-induced elevations in blood NLR, recruitment of PF neutrophils and NLR, and greater splenic contraction compared with males (IM and CM). However, it several select immune measurements were greater in males such as IL1β and CCL2. The female-biased immune reactivity observed in this study is in agreement with the literature on sex differences in humans and rodent models. This is further supported by our previous studies showing that EW-F pigs exhibited higher CRP levels^7^, enhanced intestinal mast cell activation and mediator release, and intestinal barrier permeability^9^ compared with LW-F and CM. Why EW-F exhibit a heightened immune response remains unknown. Female-biased immune activation has often been ascribed to the impacts of adult gonadal sex hormones^11^. For example, estrogen is well known to exert multifaceted effects on the immune system and contribute to sex differences^17^. Depending on its concentration, the immune cell type involved, and the stage of the immune response, estrogen can activate and/or modulate immune responses by promoting the proliferation and activation of immune cells such as B and T lymphocytes and the production of inflammatory cytokines^17–20^. However, in the current study pigs were at a prepubertal stage and thus had low levels of gonadal estrogen. Therefore, female gonadal hormones are unlikely to play a major role in this observed sex difference. One plausible mechanism could be the existence of immune-related genes on the X chromosome. The X chromosome harbors a multitude of genes involved in immune function and given that females possess two copies versus one in males, this could confer a genetic advantage, thereby contributing to the enhanced immune response. Another potential factor could be the heightened stress reactivity in females^21–25^. Previous studies have suggested that females may exhibit increased sensitivity to stress, which when compounded with early-life stressors, could potentiate the immune response^26^.

As discussed above, EW-F pigs exhibited heightened neutrophil responses, both in the blood and peritoneal fluid, following the 4 h LPS challenge compared with LW-F. However, EW appeared to impact IM in a distinctly different pattern. Opposite to F, EW in IM resulted in suppressed neutrophil recruitment to the PF in response to LPS compared to LW IM, suggesting EW might negatively influence neutrophil trafficking in IM. Irrespective of their wean age, CM showed a markedly suppressed neutrophil migration to the PF compared with other groups. This pattern was observed despite significant LPS-induced alterations in CBC and elevated cytokines in the blood. This suggests a potential defect or delayed immune cell migration in CM and EW IM. While the mechanism is currently unclear for this response, it is worth considering the potential role of neonatal gonadal androgens in males. During prenatal life and shortly after birth, the male testes produce significant amounts of androgen (testosterone and estrogen) resulting in elevations in circulating testosterone and estrogen in early life^27^. While the perinatal androgen surge in males is best known for its role in masculinizing effects across many body systems, they also are important regulators of the HPA stress axis and immune regulation in males. Thus, the suppression of gonadal androgens in male pigs via castration, or via EW stress and LPS challenge in EW-MI pigs, could have significant implications for male immune development and stress resiliency. Testosterone via androgen receptors is critical for immune cell migration towards inflammation site and regulation of neutrophil production^28^. Thus, reduced or depleted testosterone levels in EW IM and CM could impair these parameters, leading to an attenuated neutrophil response as observed in this study. In support of this, CM and EW IM groups exhibited diminished numbers of band neutrophils and reduced recovery of neutrophil numbers between 2 and 4 h post LPS challenge, indicative of a reduced capacity for neutrophil production in response to an inflammatory stimulus. Coupled with potential impairment in neutrophil migration due to low testosterone, this might contribute to the lower neutrophil counts observed in the PF of these groups. Together, early-life stressors such as EW and physiological stressors like castration can exert substantial influences on the immune system, possibly leading to immune dysregulation. In the case of EW IM and CM, this dysregulation manifests as diminished neutrophil responses to LPS challenge, which could have significant implications for host defense mechanisms. Given the crucial role of neutrophils in the initial immune response to bacterial infections, a reduction in neutrophil counts may increase susceptibility to infections, especially those caused by gram-negative bacteria that produce LPS.

We investigated whether EW or biological sex impacted adaptive immune responses using PCV2 vaccination-induced IgG response as a model. Stratifying results based on weaning age and sex revealed significant effects for each variable and time post-vaccination. Interactions between weaning age and sex, weaning age and time, and sex and time were observed, suggesting a complex interplay between these factors. Early-weaned females and EW-IM had higher PCV2 vaccine-specific IgG titers compared to LW-F and LW-IM, respectively, suggesting that EW augmented vaccine-induced adaptive immune response in these sexes. Different response responses were observed in CM which was dependent on weaning age. Among the LW pigs, LW-CM had higher titers compared to LW-F and LW-IM pigs, which is consistent with the role of androgens as negative regulators of B cell production. Supporting this, LW-CM also showed larger spleen size, indicating increased immune activity. However, the response in EW pigs differed. In EW-CM, there was a contrasting outcome compared to EW-F and EW-IM, with a suppressed IgG response relative to LW-IM. The exact reason why EW led to this suppressed response remains unclear, but it suggests that EW may have a negative impact on a specific aspect of the vaccine-induced IgG response. These findings underscore the intricate relationship between early life stressors, sex, and immune function, encompassing both innate and adaptive immunity.

The present study highlighted the complex relationship between early weaning, cortisol responses to LPS challenge, and sex effects. Early weaned intact males exhibited elevated cortisol responses, while castrated males and females did not. This suggests that the impact of early weaning on the HPA axis, varies depending on sex and gonadal status. Elevated cortisol levels can suppress the immune system, impairing immune response to challenges like LPS. The sex-specific cortisol response aligns with previous research showing sex-specific increases in HPA axis responses due to early life adversity. Testosterone, a negative regulator of the HPA axis^29^, may play a role in this context. Our study found a greater decline in testosterone levels in early weaned intact males compared to later-weaned intact males, suggesting that early weaning disrupts testosterone's regulation of the HPA axis. This disruption could contribute to the sex-specific cortisol responses observed. Additionally, the absence of increased cortisol response in early-weaned castrated males suggests that gonadal hormones, including testosterone, may modulate the stress response to early weaning.

Metabolic regulation of the immune response is critical to the overall energy management and functionality of immune cells. Immune cells primarily use glucose as their energy substrate; therefore, the availability of this metabolite directly correlates with the robustness and efficiency of the immune response. In the present study, wean age and sex had significant impacts on metabolic markers of glucose, insulin, and NEFAs following LPS challenge. In general, LPS induced a hypoglycemic response in all pigs which is largely due to increased uptake and utilization of glucose, the primary fuel source for activated immune cells. However, EW-F pigs, who displayed the greatest immune responses to LPS, had a relatively attenuated hypoglycemic responses compared with LW-F pigs. This was unexpected, as EW-F exhibited the greatest immune response to LPS which would conceivably demand substantial energy and glucose resources. The diminished hypoglycemic response in EW-F pigs could be a regulatory mechanism to maintain sufficient glucose levels during such periods of intense immune activity. Glycogen, the main form of glucose storage, supplies a rapid source of glucose during periods of enhanced metabolic demand such as an immune response. Though the liver is a primary site for glycogen storage, immune cells also maintain their own glycogen reserves, essential under conditions of low glucose availability due to increased metabolic demand^30,31^. Another consideration is that EW-F may be able to uptake glucose more efficiently. Our prior research demonstrated that EW-F pigs exhibit enhanced GLUT2-mediated glucose transport and elevated blood glucose levels^7^. The potential for a metabolic switch to an alternative energy source in EW females is another potential explanation for enhanced ability to manage the demands of an intense immune response in EW-F pigs. In line with glucose response, insulin responses to LPS were also greatest in EW-F pigs compared with LW-F and males. The heightened insulin levels and attenuated hypoglycemic responses in EW-F pigs could reflect an insulin resistant state. Insulin resistance in adipose and muscle could increase the availability of glucose to fuel the heightened immune response in EW-F and maintain glucose levels. In summary, the heightened immune response seen in EW-F might necessitate considerable energy and glucose. Thus, the blunted hypoglycemic response might serve as an adaptive mechanism to ensure adequate glucose levels during periods of intense immune activity, especially vital given the primary role of glucose and glycogen as energy sources for immune cells. LPS incites lipolysis and circulates NEFA concentrations, a direct result of triglyceride hydrolysis in adipose tissues^32^. Here we showed that EW-IM pigs demonstrated a significantly greater level of circulating NEFA levels compared to LW-IM pigs. In addition, high NEFA levels were observed in EW-F pigs, but the trend did not achieve statistical significance (*P* = 0.10). As we have previously demonstrated, EW pigs tend to have increased adipose depots, which could contribute to the elevated NEFA levels. Furthermore, insulin resistance may contribute to these findings as insulin is known to inhibit lipolysis. Early life adversity has been associated with metabolic diseases and obesity; a correlation supported by studies using rodent models. The ability to release NEFAs may represent an adaptive mechanism in EW animals, favoring energy storage in visceral adipose tissue to support heightened immune responses. Together, these results provide further insights into the intricate relationship between early life experiences, metabolic responses, and immune function, suggesting potential adaptations and implications for long-term health outcomes.

In summary, this study demonstrates the important roles that weaning age, biological sex, and castration status play in shaping the neuroendocrine and immune responses in pigs in later life. These studies uncovered that EW induces a trajectory towards long-term, heightened immune responses in female pigs, encompassing both systemic and local innate and adaptive immunity. In contrast, the immune response in EW IM or CM pigs presented an intriguing dichotomy, triggering local immune suppression. The interplay between metabolic, hormonal, and immune responses brought to light in our study suggests that metabolic or hormonal shifts may underpin the effects of weaning age and sex on programming of the immune system. These findings emphasize the necessity of considering factors such as weaning age, biological sex, and castration when formulating strategies to enhance health and productivity in pigs. Furthermore, our research has implications beyond animal health and production. The parallels between pigs and human immune systems mean that these findings could provide insightful translational perspectives that could inform the pathophysiology of early life adversity-associated outcomes in people. By understanding how early life experiences shape immune responses later in life, developing more effective strategies to bolster health across species could be possible.

### Supplementary Information

Supplementary Figure 1.

Supplementary Tables.

## Data Availability

The data supporting the findings of this study are available upon reasonable request. Researchers interested in accessing the data can contact the corresponding author [Adam Moeser, moeserad@msu.edu] to discuss the availability of the data and any necessary requirements for data access. The data will be made available in accordance with ethical considerations and data sharing policies, ensuring the protection of participant confidentiality and compliance with relevant data protection regulations.
